# Effects of exogenous testosterone application on network connectivity within emotion regulation systems

**DOI:** 10.1038/s41598-020-59329-0

**Published:** 2020-02-11

**Authors:** Mikhail Votinov, Lisa Wagels, Felix Hoffstaedter, Thilo Kellermann, Katharina S. Goerlich, Simon B. Eickhoff, Ute Habel

**Affiliations:** 1 Department of Psychiatry, Psychotherapy and Psychosomatics, Medical Faculty, RWTH Aachen University, 52074 Aachen, Germany; 2 Institute of Neuroscience and Medicine (INM-7), Research Centre Jülich, Jülich, Germany; 3 Institute of Neuroscience and Medicine (INM-10), Research Centre Jülich, Jülich, Germany; 4 Institute of Systems Neuroscience, Medical Faculty, Heinrich Heine University Düsseldorf, Düsseldorf, Germany; 5 Department of Biomedical Sciences of Cells & Systems, Section Cognitive Neuroscience, University Medical Center Groningen, University of Groningen, Groningen, The Netherlands

**Keywords:** Cognitive control, Amygdala, Prefrontal cortex

## Abstract

Studies with steroid hormones underlined the vital role of testosterone on social-emotional processing. However, there is still a lack of studies investigating whether testosterone modulates network connectivity during resting-state. Here, we tested how the exogenous application of testosterone would affect functional connectivity between regions implicated in emotion regulation. In total, 96 male participants underwent resting-state fMRI scanning. Before the measurement, half of the subjects received 5 g Testim^TM^ gel (containing 50 mg testosterone) and the other half a corresponding amount of placebo gel. Seeds for the connectivity analysis were meta-analytically defined. First, all regions associated with emotion regulation were chosen via Neurosynth (data driven). Among those, specific seeds were selected and categorized based on the neural model of emotion regulation by Etkin and colleagues (Etkin *et al*., 2015) (theory-guided). Resting-state connectivity analysis revealed decreased connectivity between the right DLPFC and the right amygdala as well as between the VMPFC and the left IPL for the testosterone group compared to the placebo group. A complementary dynamic causal modeling (DCM) analysis on findings from the resting-state connectivity analysis underlined a bidirectional coupling which was decreased close to zero by testosterone administration. Our results demonstrate that testosterone administration disrupts resting-state connectivity within fronto-subcortical and fronto-parietal circuits. The findings suggest that even without a specific task (e.g. challenge, reward processing) testosterone modulates brain networks important for social-emotional processing.

## Introduction

Gonadal hormones play a significant role in the modulation of the human emotion circuit^1^. For a long time, the male sex hormone, testosterone, has been primarily associated with socially negative behaviors such as aggression^2^ and risk-taking^3^. Recent studies point towards a more complex role of testosterone during human interactions, suggesting that it also affects positive emotions^4^ and can promote cooperative and prosocial behavior^5,6^. Further, it has been speculated that advantageous skills in cognitive empathy of females may be a result of lower testosterone levels^7^. While research evidently demonstrated that the social context has a decisive influence on the direction of the testosterone effect^6^, the neural mechanisms associated with this contextually mediated hormonal effect are still far from understood.

In humans, the influence of exogenous testosterone on emotion and behavior has been linked to functional alterations of several brain structures. Studies measuring the endogenous level of testosterone (*natural physiological variations*) revealed that particularly amygdala activity seems to be affected by circulating testosterone levels among females and males^8–11^. The influence of exogenous testosterone (*artificial hormonal manipulation; usually transdermal or oral administration*) on emotion and behavior has been linked to functional alterations of several brain structures. Testosterone administration in humans modulates, for instance, striatal activity during reward processing in women^12^, insular activity during risk-taking in men^13^, and amygdala activity in women when responding to social threats^14,15^. Furthermore, modulated amygdala−prefrontal coupling was observed after testosterone administration in both genders and with changing endogenous testosterone levels in puberty^1,16–19^. On the whole, these studies point towards a testosterone-induced reduction of regulatory control (exerted by the prefrontal cortex) over the amygdala^1,17,18^.

The first studies in humans that established an important role of testosterone on functional connectivity were performed in the field of electrophysiology^20,21^. A decoupling of midfrontal delta-beta oscillations was linked to the disinhibitory properties of testosterone administration^20^. FMRI studies provided further evidence for a modulatory role of testosterone on brain connectivity. In women, reduced cortico-subcortical connectivity after testosterone administration was observed during a face-matching task^1^. A further study also applying a face-matching task found that testosterone administration reduced the connectivity between the inferior frontal gyrus and the supplementary motor area in female participants^7^. While findings on the effect of a single dose of testosterone on resting-state connectivity are currently lacking, there is some indication of a potential effect provided by the investigation of male anabolic steroid users. In those, modulated connectivity between key areas of the emotion regulation system and large-scale brain networks was observed, compared to past users and to controls. Specifically, the researchers found reduced functional connectivity between the superior frontal gyrus and the dorsal attention network as well as between the amygdala and the default mode network^22^. Another study found that when facing an acute threat, a single testosterone administration decoupled the left lateral orbitofrontal cortex from a subcortical system including the central-medial amygdala, hypothalamus, and periaqueductal gray^23^.

Overall, results from previous studies converge towards an essential role of testosterone on social-emotional processing and emphasize the involvement of specific target regions such as the amygdala. However, previous studies mainly focused on task-related differences in neural activation and connectivity in relation to testosterone and its influence on behavioral changes. Despite the well-established role of testosterone during social contexts, the influence of exogenous testosterone on neural connectivity during rest is currently not well-understood.

**Figure 1:**
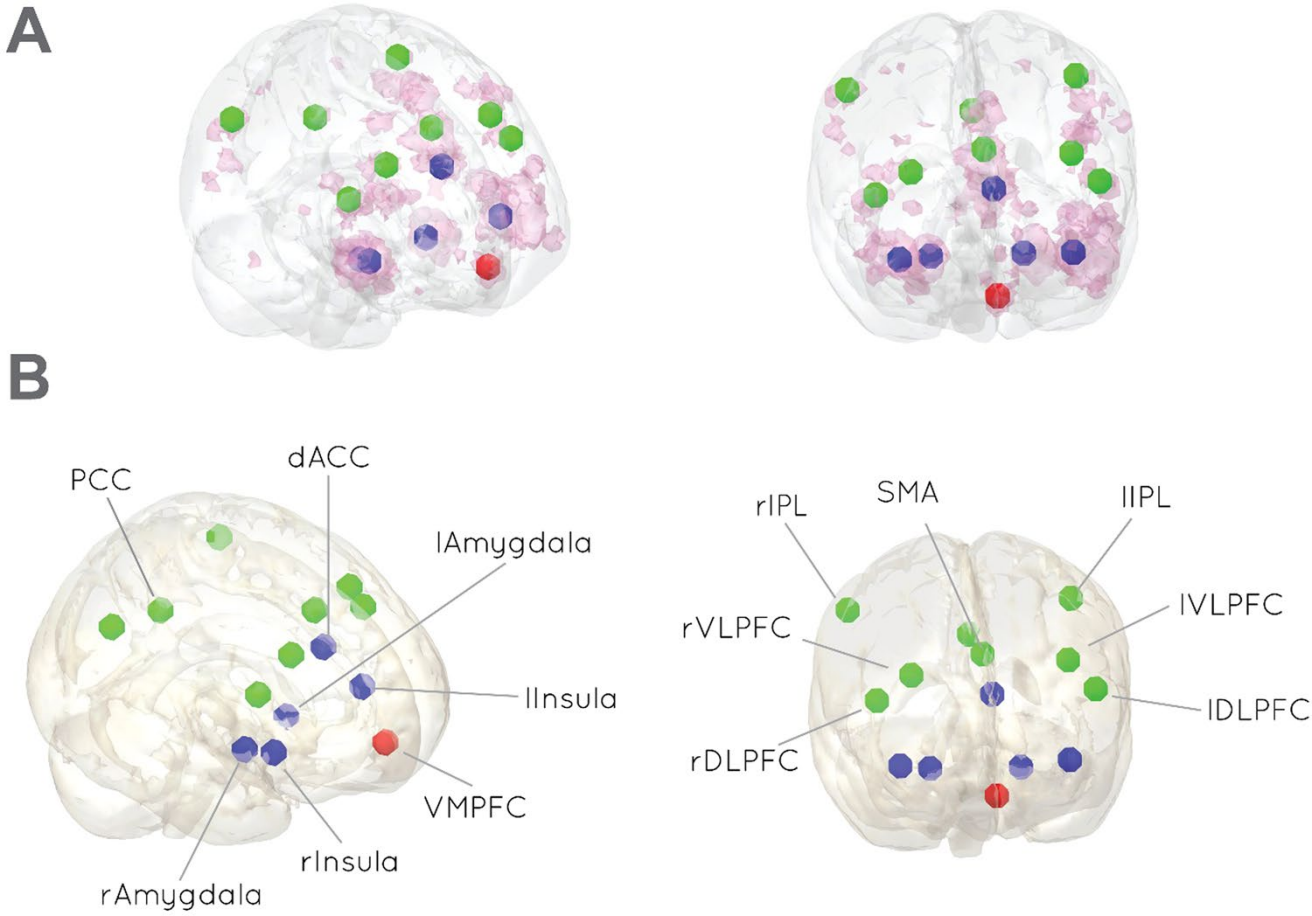
Regions of interest were defined in a two-pass procedure. (**A**) Activation map (pink color) was obtained by conducting a neuroimaging meta-analysis for the term “emotion regulation” in Neurosynth (161 fMRI studies). (**B**) Only ROIs that were given by the Etkin model was selected as seed regions. Fourteen 5 mm sphere ROIs which matched with the three systems: blue color -perception (left and right amygdala, insula, and dACC), red color - valuation (VMPFC) and green color - action (DLPFC, inferior parietal lobule (IPL), SMA, VLPFC, PCC).

Here, we investigated the role of exogenous testosterone on resting-state connectivity within the framework of the emotion regulation model by Etkin and colleagues^24^. This model postulates that emotion regulation comprises all processes that are used to initiate, stop, or modulate a certain emotion^25^. As a complex process, emotion regulation in the model of Etkin and colleagues is assumed to encompass several systems that have specific functions, such as emotional reactivity (perception), evaluation of the need for regulation (valuation), and regulatory actions (action)^24^. Being described as three specific neural systems, each process corresponds to a set of common brain regions (Fig. 1). The first system, emotional reactivity, includes the dorsal anterior cingulate (dACC), the insula, the amygdala and the periaqueductal grey (PAG). Evaluating the need for emotion regulation is reflected in ventromedial prefrontal (VMPFC) activation, the second system, whereas regulatory actions are thought to be mediated by the third system, represented by the dorsolateral (DLPFC) and ventrolateral prefrontal cortices (VLPFC), supplementary motor area (SMA), pre-SMA and parietal cortex. How these systems communicate depends on the regulation strategy, which is either model-based, applying internal rules and relying on regulatory actions, or model-free, where the need for regulation is flexibly adapted by evaluating contextual changes. Testosterone might induce connectivity changes among these systems.

By investigating resting-state brain connectivity of regions involved in emotion regulation, this study aimed to reveal the effects of testosterone on network connectivity during rest in human males. We hypothesized that a single application of exogeneous testosterone would modulate resting-state connectivity in the specified emotion regulation network. Based on the results of functional studies, we specifically predicted a testosterone induced reduction of cortico-subcortical connectivity.

## Methods

### Sample

In total, 96 healthy males underwent resting-state scanning. Women were not included because in Germany, Testim^TM^ (the applied testosterone gel) is currently not approved for use in women. According to the Edinburgh Handedness Inventory (Oldfield 1971), all participants were right-handed. All participants had normal or corrected-to-normal vision, no MR contraindications, no history of traumatic brain injury, and no psychiatric or neurological illness in the present or past.

### Procedure

All participants gave oral and written informed consent prior to the study. They received a fixed financial compensation of 70 Euro for participation, and could additionally win between 15 and 35 Euro depending on individual performance during the Technical Provocation Paradigm^26^ and the balloon analogue risk task^13^. Experimental procedures were approved by the Ethics Committee of the Medical Faculty of the RWTH Aachen University and were in accordance with the Helsinki declaration (1964) and its later amendments or comparable ethical standards.

Data acquisition always started in the early afternoon (between 12.00 and 14.00). During the first part of the experiment (Time 1) the first blood sample was taken (baseline cortisol and testosterone levels), and participants filled in several questionnaires assessing individual differences in trait measures related to emotion, impulsivity, and aggression, and participants completed brief behavioral tasks. The application of the 5 g Testim^TM^ gel corresponding to 50 mg testosterone and the placebo gel, respectively, followed directly after taking the first blood sample (Time 1) and was carried out in a randomized, double-blind manner. The gel was applied to the skin of the upper back and shoulders of the participants (for more details, see Wagels and colleagues^27^). After the gel application, the participants completed the behavioral tasks and filled in the questionnaires. A second blood sample (Time 2) was collected right before the participants went into the scanner, approximately three and half hours after testosterone/placebo administration. A further blood sample (Time 3) was collected in the scanner room after the first fMRI task^13^; for this, the participant was taken halfway out of the scanner. After the second (and last) fMRI task, the last blood sample (Time 4) was taken. Finally, the resting-state scan, lasting five minutes, and an anatomical scan were performed. During the resting state scan, participants lay supine in the scanner with their eyes open facing a fixation cross, instructed to relax without falling asleep.

### Questionnaires

Participants filled in the Barratt Impulsiveness Scale (BIS-11)^28^, the Buss and Perry Aggression Questionnaire^29^ and the Psychopathic Personality Inventory (PPI)^30^. Also, the Emotion Regulation Questionnaire (ERQ) and PANAS (Positive And Negative Affective State) scale were applied^31,32^. Group differences in impulsivity, aggression and psychopathy scores were assessed using MANOVAs with group (testosterone versus placebo) as a between-subject factor and total scores and subscale scores from the respective questionnaire as dependable variables. In case of violations of the sphericity assumption, Greenhouse Geisser correction was applied. Results for post-hoc tests were based on a Bonferroni adjusted alpha level.

### Genetic and hormonal assessment and analysis

The polymorphism of the monoamine oxidase A genotype categorized in MAOA-L (long tandem repeats) and MAOA-S (short tandem repeats) variants was determined prior to the study (for full details, see supplementary data). The MAOA polymorphism was included as a covariate in all statistical analyses, because hormonal effects can be modulated by genetic manifestation regarding the MAOA polymorphism^13,33–36^.

Testosterone levels were assessed by the laboratory (Labordiagnostisches Zentrum Uniklinik Aachen) via blood serum samples at four time points during the study. Samples were analyzed using an immunologic *in vitro* quantitative determination of testosterone (Electrochemiluminescence immunoassay, ECLIA; RocheVR Diagnostics GmbH, Mannheim, Germany, www.roche-diagnostics.com). The functional sensitivity and specificity of the applied method was 0.087–52.0 nmol/L. Intra- and interassay variability of testosterone was below 3%. A two-way repeated-measures ANCOVA was performed comparing testosterone levels as a function of time by hormone application including age and genotype as covariates. The within-subject factor time had four levels: the baseline prior to hormonal treatment (Time 1), the serum levels prior to the scanning session (Time 2), the time point between the two fMRI tasks (Time 3), and the time point just before the resting-state scan (Time 4). Between subject factors were treatment group (testosterone, placebo), and covariates were genotype (MAOA-S, MAOA-L) and age. In case of violations of the sphericity assumption, Greenhouse Geisser corrections were used. Results for post-hoc tests were based on a Bonferroni adjusted alpha level.

### fMRI data acquisition and analyses

Imaging data were acquired using a Siemens 3 T Prisma scanner (Siemens AG; Erlangen, Germany) equipped with a 12-channel head matrix coil located in the Department of Psychiatry, Psychotherapy, and Psychosomatics, RWTH Aachen University. The resting-state measurement was performed as part of a longer scan session that additionally included two fMRI tasks and an anatomical scan. Resting-state MRI data were obtained using echo-planar imaging with the following scanning parameters: TR = 2000 ms; TE = 30 ms, flip angle = 77°, FOV = 192 × 192 mm², matrix size = 64 × 64 mm, 36 slices, slice thickness = 3.1 mm, volumes = 215, voxel size = 3 × 3 × 3 mm³, interleaved ascending, slice gap 1 mm.

### Creating regions of interest for the emotion regulation network

The neural emotion regulation system was based on the model proposed by Etkin^24^. Regions of interest were defined by a data-driven approach and a theory-guided selection. As part of the data-driven approach, regions of interest (ROIs) were obtained by conducting a neuroimaging meta-analysis for the term “emotion regulation” in Neurosynth (http://www.neurosynth.org, see supplementary materials). Afterward, ROIs that were obtained during the meta-analysis were clustered into three specific neural systems corresponding to the Etkin model of emotion regulation (emotional reactivity system, evaluation system, and regulatory system). This meta-analytical and theoretical approach was taken for reasons of double-validation in terms of theory and data driven analysis.

The initial query found 161 fMRI studies revealing brain regions of significant association with emotion regulation tasks (false discovery rate (FDR) corrected p < 0.01^37^). Subsequently, 14 peak coordinates were derived from the selected regions (see Supplementary Table 1) representing the three systems for emotion perception (left and right amygdala, left and right insula, and dACC), valuation (VMPFC) and action (left and right DLPFC, left and right inferior parietal lobule (IPL), SMA, left and right VLPFC, and PCC (see Fig. 1).

### Resting-state analysis

Resting-state data were preprocessed by SPM12 and in-house Matlab tools. Data of each participant were preprocessed by applying FIX (FMRIB’s ICA-based Xnoiseifier) implemented in FSL (http://fsl.fmrib.ox.ac.uk/fsl/fslwiki/FSL) to remove physiological and movement artifacts.

FIX decomposes the data into independent components and automatically classifies noise components by using a large number of distinct spatial and temporal features via pattern classification^38^. We used the training datasets provided with FIX and the recommended settings^39^, which had also shown very promising results in a clinical sample^40^. Variance uniquely related to the noise components was then removed from each participant’s raw data. Slice timing correction was applied and images were corrected for head movement by affine registration using a two-pass procedure by which images were initially realigned to the first image and subsequently to the mean of the realigned images. Each participant’s data was then spatially normalized to the MNI (Montreal Neurological Institute) 152-subject average template brain, included in SPM12, using the “unified segmentation” approach^41^. The ensuing deformation was applied to all individual EPI images. Finally, images were spatially smoothed by a 5-mm radius full-width at half-maximum Gaussian kernel and band-pass filtered preserving Blood Oxygen Level Dependent (BOLD) signal frequencies between 0.01 and 0.08 Hz. For each participant, the time-series of each seed region’s BOLD signal was extracted as the first eigenvariate of activity in a 5 mm sphere around the peak coordinate from the Neurosynth analysis.

Using the FSLNets toolbox (http://fsl.fmrib.ox.ac.uk/fsl/fslwiki/FSLNets), partial temporal correlations between the time-series (eigenvariates) of all fourteen regions of interests (see coordinates in supplementary data) were computed to estimate pairwise functional connectivity (FC)^42^ corrected for age and MAOA polymorphism. Based on these Fisher’s Z–transformed FC values, we compared the network connectivities between the two groups using two-sample t-tests. Each of the three t-tests was Bonferroni corrected to control for the number of between-systems connections in that specific comparison. In other words, the group comparison for the connectivity between the emotion perception system (5 nodes) and the action system (8 nodes) was corrected for 8 × 5 = 40 connectivities, the comparison between the emotion perception system (5 nodes) and the valuation system (1 node) was corrected for 5 connectivities, and the comparison between the motor system (8 nodes) and the valuation system (1 node) was corrected for 8 connectivities. We emphasize that no differences in between-network connectivity between the groups were found when controlling for all 53 comparisons. One outlier (value over two standard deviations) was removed from the network resting-state connectivity analysis.

### Complimentary dynamic causal modelling analysis

In order to characterize the directional effects of the group differences previously observed via network resting-state analysis, we additionally applied dynamic causal modeling (DCM)^43,44^. We specified DCM only for each of the two pairs of regions (right amygdala-right DLPFC and VMPFC-left IPL) with full (i.e. reciprocal) connectivity between the respective two nodes, because only these two pairs revealed significant results in the resting-state connectivity analysis. Similar to the previous analysis, we extracted the first eigenvariate of all voxels within a sphere of a 5 mm radius around the predefined center of the respective region. Model inversion was performed using DCM10 as implemented in SPM12 (due to better performance) with the stochastic option for resting-state DCM (and one state per region). Extrinsic (between regions) coupling parameters were further analyzed in repeated measures two-by-two ANCOVAs using age and MAO-A genotype as covariates. The between subject factor was treatment group (testosterone and placebo) and within subject factors was directionality (from region A to region B and from region B to region A). One outlier was removed (value over two standard deviations).

### Correlations between functional connectivity and personality

Correlation analyses were performed only on significant FC pairs. Fisher’s z–transformed FC values of the two pairs right amygdala-right DLPFC and VMPFC-left IPL were correlated with results from the neuropsychological scales (ERQ and PANAS). Two-tailed Pearson partial correlations controlling for MAOA and age were performed for the whole sample, followed by the same analyses (one-tailed) performed separately for the placebo group (n = 45) and the testosterone group (n = 50) (p < 0.05).

## Results

### Sample and hormonal results

The testosterone and placebo groups did not significantly differ in regards to age. To exclude possible additional influencing factors, we tested for differences in trait characteristics between the groups. We did not observe any significant differences between treatment groups for the questionnaires assessed, i.e., the BIS-11, AQ, PPI, and ERQ (all total scores p > 0.2, see Supplementary Table 3). One subject from the testosterone group did not fill in the BIS-11, AQ, and the ERQ.

**Figure 2:**
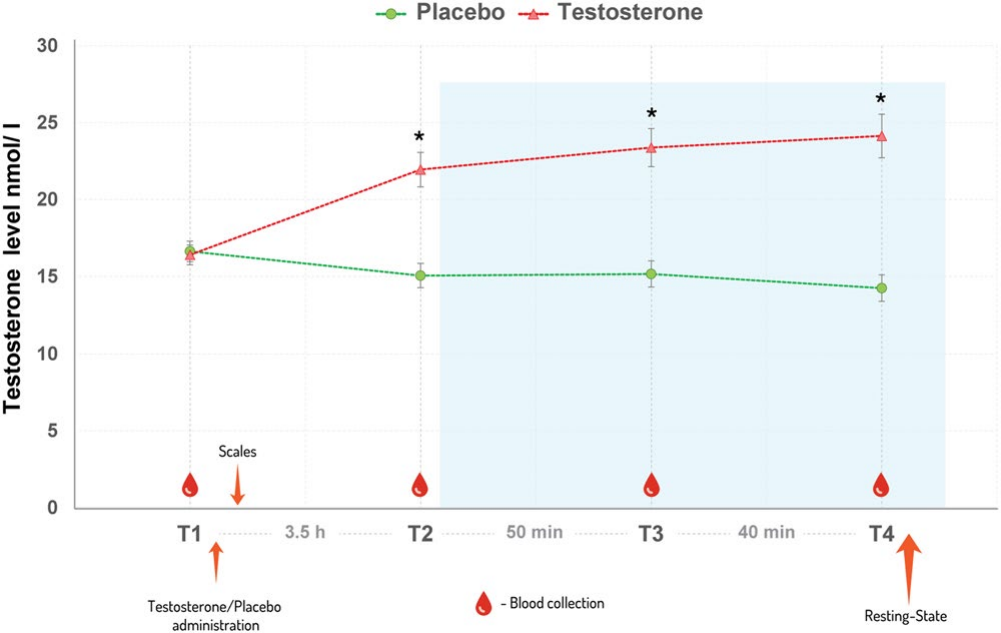
Hormone levels are presented separately for the testosterone (T) and placebo (PL) group. Mean raw levels and standard errors are indicated. Testosterone levels were significantly higher after applying the testosterone gel in the testosterone group (see time points T2, T3, and T4 indicated by *). Blue colored square represents time in the scanner.

Repeated measures ANCOVA on the 96 participants confirmed a significantly higher testosterone level after hormone application in the testosterone group compared to the placebo group. Following a non-significant difference in testosterone levels at baseline (Time 1, p = 0.81), hormonal levels differed significantly between the groups already at Time 2 and were further significant at Time 3 and Time 4 (all p < 0.001). For a complete overview of the differences in testosterone levels between the two groups at each time-point, see Fig. 2 and Supplementary Table 2.

### Network resting-state connectivity

**Figure 3:**
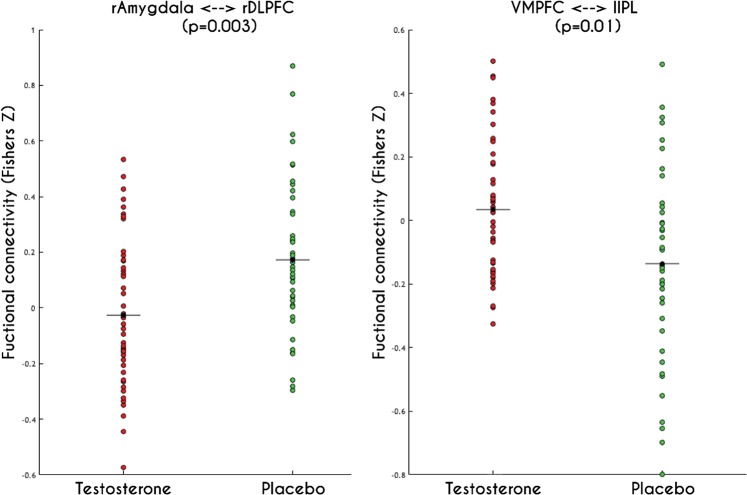
Network resting-state connectivity revealed the significant difference between groups for two pairs of the region of interests. Left: Between right Amygdala and right Dorsolateral Prefrontal Cortex (DLPFC). Right: Between Ventromedial Prefrontal Cortex (VMPFC) and left Ipsilateral Parietal Lobule (IPL).

The analysis (95 participants) revealed a significant difference between the groups for two ROI pairs only. In the testosterone group (T) compared to the placebo group (PL), reduced connectivity between the right DLPFC and the right amygdala (emotion regulation system) was observed (mean ± standard deviation (SD); PL = 0.16 ± 0.27; T = −0.021 ± 0.33; p = 0.003). In contrast stronger de-coupling was found between the VMPFC and the left IPL (action regulation system) for the placebo group compared to the testosterone group (mean ± SD; PL = −0.16 ± 0.39; T = 0.01 ± 0.29; p = 0.01; see Fig. 3).

### Complimentary dynamic causal modelling (DCM)

**Figure 4:**
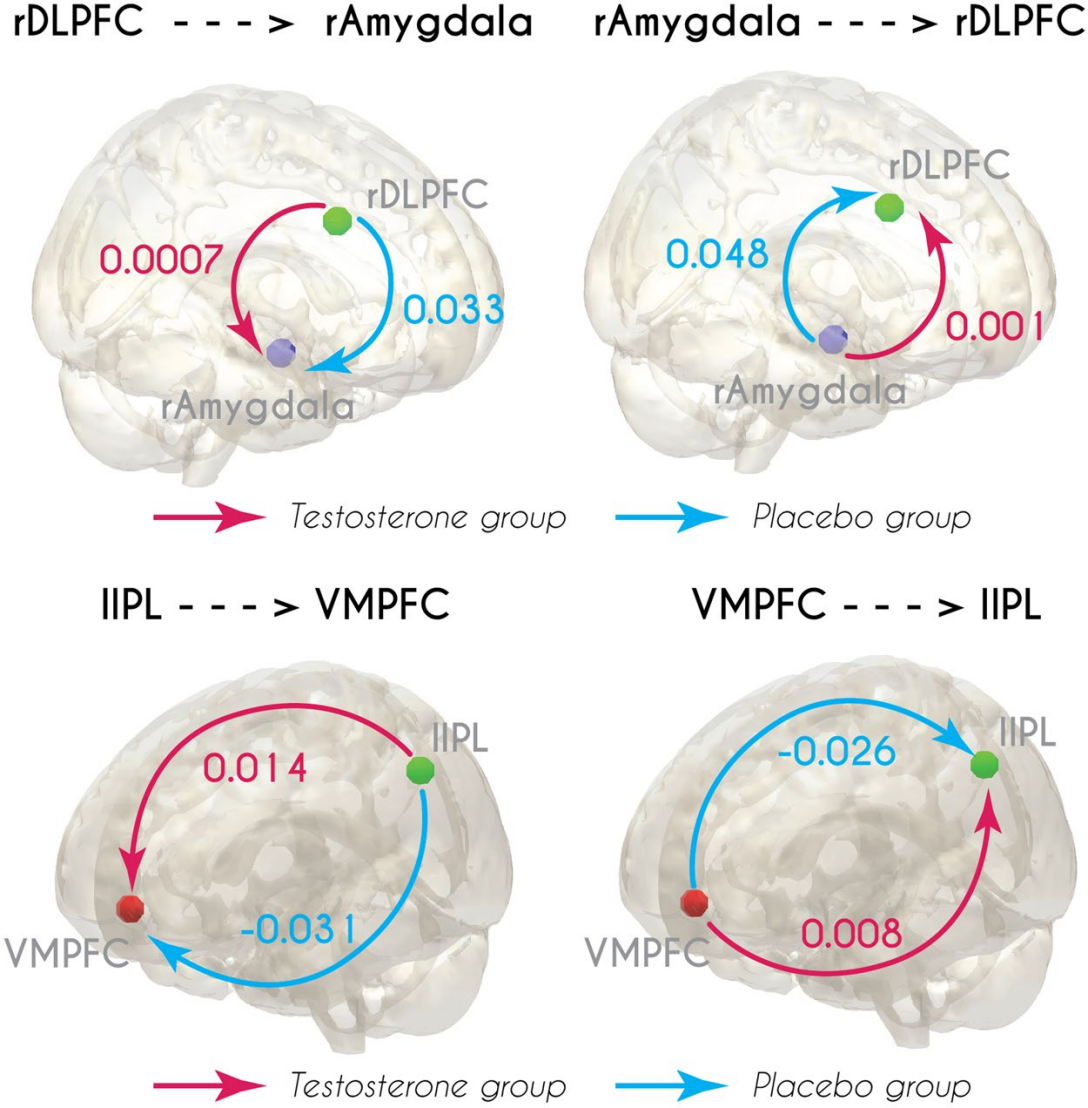
Results of the DCM analysis. Top: Directional connectivity between the right Dorsolateral Prefrontal Cortex (DLPFC) and the right amygdala was decreased in the testosterone group compared to the placebo group. Connectivity in the placebo group from the right amygdala to the right Dorsolateral Prefrontal Cortex (DLPFC) was stronger than for the reversed directionality. Bottom: Directional connectivity between Ventromedial Prefrontal Cortex. (VMPFC) and left ipsilateral parietal lobule (IPL) was decreased in the placebo group compared to the testosterone group.

The DCM analysis (95 participants) revealed that the directional connectivity between the right DLPFC and the right amygdala was lower in the testosterone group (see Fig. 4). There was a main effect of group, F(1,91) = 6.39, p = 0.013 and an interaction of direction × group, F(1,91) = 5.59, p = 0.02. In the testosterone group (T) compared to the placebo group (PL), smaller connectivity parameters were observed from the amygdala to the DLPFC (mean ± SD; PL = 0.048 ± 0.08; T = 0.001 ± 0.09; p = 0.011) as well as from the DLPFC to the amygdala (mean ± SD; PL = 0.033 ± 0.05; T = 0.007 ± 0.07; p = 0.02). Interestingly, connectivity in the placebo group from the right amygdala to the right DLPFC was stronger than for the reversed direction (p = 0.001).

The DCM analysis of the connectivity between the VMPFC and the left IPL also demonstrated significant differences between the groups (see Fig. 4). There was a significant main effect of group, F(1,91) = 7.15, p = 0.009, and an interaction of direction × group at trend level, F(1,91) = 3.12, p = 0.08. In the placebo group compared to the testosterone group, connectivity parameters were significantly more negative from the left IPL to the VMPFC (PL = −0.031 ± 0.08; T = 0.014 ± 0.08; p = 0.007), and from the VMPFC to the left IPL (PL = −0.026 ± 0.07; T = 0.008 ± 0.06; p = 0.016). Moreover, when we compared the results for the two FC pairs against zero, we observed that connectivity values in the testosterone group were not significantly different from zero.

### Correlations between functional connectivity and personality

The rAmy ↔ rDLPFC connectivity was negatively correlated to the ERQ subscale suppression (r = −0.28, p = 0.008) across the whole sample, indicating that less frequent use of emotional suppression was associated with stronger connectivity between these regions. Repeating the analysis separately for each group, we show that this association was significant both in the placebo group (r = −0.33, p = 0.019) and in the testosterone group (r = −0.26, p = 0.038). Fisher’s r-to-z calculation indicated that the difference between these two correlation coefficients was not significant (z = −0.36, p = 0.35).

Moreover, rAmy ↔ rDLPFC connectivity was negatively correlated to the PANAS subscale Negative Affect (r = −0.2, p = 0.059) across the whole sample. Separate analyses for each group showed that this association was significant both in the placebo group (r = −0.33, p = 0.018) and at trend level in the testosterone group (r = −0.22, p = 0.064). Fisher’s r-to-z calculation indicated that the difference between these two correlation coefficients was not significant (z = −0.55, p = 0.29).

## Discussion

In the current study, we tested the effects of exogenous testosterone on resting-state connectivity within the emotion regulation network suggested by Etkin and colleagues. Our findings provide new evidence that exogenous hormone application may decrease connectivity within fronto-subcortical (action-perception system) and fronto-parietal (action-valuation system) regions. We discovered that in the testosterone group, connectivity was decreased between the amygdala and the DLPFC and between the VMPFC and the IPL. Moreover, additional DCM analyses revealed that these regions were coupled in both directions and that testosterone administration decreased this coupling close to zero.

Sex steroids such as testosterone have previously been shown to modulate brain connectivity. Specifically, exogenous and endogenous levels of testosterone appear to decrease subcortical—cortical connectivity, while other findings suggest increased connectivity between subcortical brain areas (for a review, see^45^). Additionally, endogenous testosterone levels seem to modulate the coupling of the amygdala and the prefrontal cortex depending on the congruence of affective stimuli^16^ and emotional state change^19^. Functional MRI studies support the finding that exogenous testosterone modulates functional connectivity during emotion-related task performance. One study observed reduced functional coupling of the amygdala and the orbitofrontal cortex, and enhanced amygdala coupling with the thalamus^1^. Another study reported reduced connectivity between the inferior frontal gyrus and the supplementary motor area when matching emotions to faces^7^. Consequently, testosterone may reduce cortical regulatory control over the amygdala and impair emotion-recognition abilities. Importantly, fronto-limbic connectivity can also be affected by estrogen levels^46^, and it is not clear if the effects of testosterone administration are directly related to testosterone levels or interactions with other sex-hormones. Both above-mentioned studies were conducted within young female participants only. Our present results extend this line of evidence by demonstrating that testosterone reduced frontolimbic and frontoparietal connectivity during rest in men. Previously, indirect evidence for the effect of testosterone came from a resting-state study in male anabolic steroid users who engaged in heavy resistance strength training^22^ reporting reduced coupling of the dorsal attention network with the superior frontal gyrus, as well as decreased coupling of the default mode network with the amygdala in comparison to a control group^22^. Moreover, a study by Heany *et al*.^23^ showed that testosterone activated the left lateral OFC in situations of goal-directed escape anticipation, and decoupled the left lateral OFC from a subcortical threat network (i.e. central-medial amygdala, hypothalamus, and PAG) during threatening situations.

The networks that were chosen as regions of interest in this study refer to a theoretical model on emotion regulation proposed by Etkin and colleagues. Based on findings from previous research on emotion regulation, their unified conceptual framework combined different brain regions in three subsystems involved in the regulation of emotions^24^. As also supported by comprehensive neuroimaging meta-analyses, emotion processing and regulation engage several frontal and subcortical brain areas. Certainly, these regions also subserve a range of emotional and cognitive processes. However, largely overlapping with the theoretical model of Etkin and colleagues, meta-analytic evidence suggests an involvement of the amygdala, DLPFC, VMPFC, aMCC/dACC, superior temporal gyrus, angular gyrus and pre-SMA/SMA in emotion regulation^47–50^. The majority of these regions have a high density of receptors for steroid hormones or aromatase enzyme, especially the amygdala^51^. The amygdala has explicitly been suggested to be involved in the application of emotional reappraisal strategies. A meta-analytical imaging study showed that the emotion regulation strategy of cognitive reappraisal activated cognitive control regions and lateral temporal cortex and modulated activity in the bilateral amygdalae^52^. Moreover, the bilateral amygdalae and parahippocampal gyrus exhibited decreased activity in down-regulated states, but an activation increase in up-regulated states^48^. Instead, cortical regions including the superior frontal gyri, cingulate, and premotor areas, showed enhanced activity across all emotion regulation conditions.

In the framework of the Etkin model, we observed decreased connectivity between the subsystems for emotional reactivity (right amygdala) and explicit emotion regulation (right DLPFC) within the testosterone group. On a neural level, a reduced top-down control from prefrontal cortices to the amygdala has been linked to a reduced application of emotional reappraisal as shown in the studies from Banks *et al*. and Kanske *et al*.^53,54^. Concerning the placebo group, our results of the DCM analysis showed that connectivity from the right amygdala to the right DLPFC was stronger than vice versa and might, therefore, indicate a stronger bottom-up process. In contrast, in the testosterone group, this connection seemed to be disrupted in both directions. Indeed, connectivity did not differ from zero. While emotion regulation strategies and neural connectivity patterns might be inherently different in resting-state compared to task-related functional connectivity, these results suggest that testosterone modulated connectivity in the emotion regulation network even during resting-state.

Our DCM analysis also revealed reduced coupling between the VMPFC and the left IPL following testosterone application. According to Etkin *et al*. 2015, the VMPFC is involved in implicit emotion regulation, and the IPL (including supramarginal and angular gyri) is engaged in the explicit execution of emotion regulation^47^. Previous human studies highlighted the role of this region in goal-directed decision-making, preference choice and value computation^55–57^. Moreover, patients with lesions in the VMPFC showed impairments in conflict adaptation during an emotional task^58^. As a key region for implicit emotion regulation, the VPMFC might signal the need for regulation to other areas such as the IPL, which then executes regulatory actions^47^. Furthermore, the recent meta-analytical study by Hiser at al. 2018 established the manifold role of the VMPFC region^59^. They identified distinct subregions within the VMPFC that correspond to three facets of functions: the reward- and value-based decision making, the generation and regulation of negative emotion, and role in multiple aspects of social cognition, such as facial emotion recognition, theory-of-mind ability, and processing self-relevant information.

Interestingly, rAmy ↔ rDLPFC connectivity also correlated negatively with the ERQ subscale suppression in both groups, i.e. irrespective of testosterone application. Thus, high trait values of emotion suppression seem to be associated with a decreased negative coupling. Comparable results were observed for the correlation with Negative Affect subscale in the PANAS questionnaire. Based on these findings, we conclude that this region-to-region connectivity is conceptually related to emotion processing and regulation. Indeed, the connectivity may reflect a sub-component of emotion regulation that is often seen as a less optimal strategy, i.e. emotion suppression.

Importantly however, this does not equal an improvement or increased use of emotion suppression via testosterone. Emotion suppression in our findings is not affected by testosterone administration which might be due to the nature of the measurement. We would not assume a trait to be affected by testosterone administration and we did not assess potential changes of emotion regulation strategies before and after testosterone administration. Thus, our results may rather demonstrate that testosterone affects neural connections that are likely used for emotion processing and regulation and probably also for aggression-related cognition. As indicated by numerous findings of increased angry, aggressive and impulsive behavior following testosterone administration^2,26,27,60,61^, alterations in regions important for emotion and cognition might be one underlying neural mechanism of such testosterone-induced changes in behavior.

The present evidence for a modulatory role of testosterone on resting-state connectivity in the emotion regulation network extends previous studies suggesting an impact of testosterone on social-emotional behavior in the context of aggression and mood disorders such as depression and anxiety^2,62^.

Patients with hypogonadism and hypoandrogenism develop depressive symptoms related to testosterone levels^63,64^, and testosterone replacement in depression has been shown to have an antidepressant effect^65^. Enhanced suppression of anxiety and sadness was demonstrated across animal studies and studies with healthy and hypogonadal men^62,66,67^. Our present findings contribute to the understanding of how testosterone modulates behavioral responding. Extending previous observations of testosterone-induced modulation of affective face processing, the current study demonstrates that testosterone alters network connectivity in emotion regulation systems already during rest, suggesting testosterone-induced alterations of basic mechanisms. Emotion regulation capabilities thus seem to be related to circulating testosterone, implying that in the case of low testosterone levels, the exogenous application may facilitate emotional regulation and the regulation of stress responses.

### Limitations

There are several limitations of this study. Considering differences in hormonal levels due to sex and age, further studies should investigate the effect of exogenous testosterone on women and in individuals of older age. There are a few technical limitations. One is the rather short resting-state measurement; the recommended time should be between 9 and 12 minutes^68^. However, even with shorter time measurement (3 minutes) observable effects of hormonal application on resting-state can be found^69^. Another one is the filtering out high-frequency noise, since it may potentially remove the meaningful signal. One more limitation is that our study did not measure emotion regulation *per se* and we used the regions from a theoretical model^24^. In order to specify the effects of testosterone on emotion regulation, future studies are needed that explicitly test how testosterone application affects emotion regulation and the underlying brain networks. Moreover, future studies should examine the effects of testosterone application with different dosages and also with resting-state measurement only.

## Conclusion

Overall, our results demonstrate that exogenous application of testosterone modifies resting-state connectivity within neural systems related to emotion regulation. Specifically, connectivity signals between the DLPCF and the amygdala as well as between the VMPFC and IPL are hampered by testosterone. These findings provide new insights into the role of testosterone in reducing fronto-subcortical and fronto-parietal connectivity potentially contributing to impaired emotion processing and regulation.

## Supplementary information

Supplementary data.

## References

[1] van Wingen G, Mattern C, Verkes RJ, Buitelaar J, Fernández GN (2010). Testosterone reduces amygdala-orbitofrontal cortex coupling. Psychoneuroendocrinology.

[2] Carre JM, McCormick CM, Hariri AR (2011). The social neuroendocrinology of human aggression. Psychoneuroendocrinology.

[3] Stenstrom E, Saad G (2011). Testosterone, financial risk-taking, and pathological gambling. J. Neurosci. Psychol. Econ..

[4] Dashjav E., Lipińska-Chwałek M., Grüner D., Mauer G., Luysberg M., Tietz F. (2016). Atomic layer deposition and high-resolution electron microscopy characterization of nickel nanoparticles for catalyst applications. Surface and Coatings Technology.

[5] Reimers, L. & Diekhof, E. K. Testosterone is associated with cooperation during intergroup competition by enhancing parochial altruism. *Front. Neurosci*. **9** (2015). 10.3389/fnins.2015.00183 PMC4464174 26124701

[6] Dreher J-C (2016). Testosterone causes both prosocial and antisocial status-enhancing behaviors in human males. Proceedings of the National Academy of Sciences.

[7] Bos PA (2016). Testosterone reduces functional connectivity during the ‘Reading the Mind in the Eyes’ Test. Psychoneuroendocrinology.

[8] Derntl B (2009). Amygdala activity to fear and anger in healthy young males is associated with testosterone. Psychoneuroendocrinology.

[9] Buades-Rotger, M. *et al*. Endogenous testosterone is associated with lower amygdala reactivity to angry faces and reduced aggressive behavior in healthy young women. *Sci. Rep*. **6** (2016). 10.1038/srep38538 PMC5141420 27924836

[10] Varkevisser T, Gladwin TE, Heesink L, van Honk J, Geuze E (2017). Resting-state functional connectivity in combat veterans suffering from impulsive aggression. Soc. Cogn. Affect. Neurosci..

[11] Buades-Rotger M, Engelke C, Krämer UM (2019). Trait and state patterns of basolateral amygdala connectivity at rest are related to endogenous testosterone and aggression in healthy young women. Brain Imaging Behav..

[12] Hermans EJ (2010). Effects of exogenous testosterone on the ventral striatal BOLD response during reward anticipation in healthy women. NeuroImage.

[13] Wagels Lisa, Votinov Mikhail, Radke Sina, Clemens Benjamin, Montag Christian, Jung Sonja, Habel Ute (2017). Blunted insula activation reflects increased risk and reward seeking as an interaction of testosterone administration and the MAOA polymorphism. Human Brain Mapping.

[14] Hermans EJ, Ramsey NF, van Honk J (2008). Exogenous testosterone enhances responsiveness to social threat in the neural circuitry of social aggression in humans. Biol. Psychiatry.

[15] Radke S (2015). Testosterone biases the amygdala toward social threat approach. Science advances.

[16] Volman I, Toni I, Verhagen L, Roelofs K (2011). Endogenous testosterone modulates prefrontal–amygdala connectivity during social emotional behavior. Cereb. Cortex.

[17] Spielberg JM (2014). Pubertal testosterone influences threat-related amygdala–orbitofrontal cortex coupling. Soc. Cogn. Affect. Neurosci..

[18] Chen C, Decety J, Huang PC, Chen CY, Cheng Y (2016). Testosterone administration in females modulates moral judgment and patterns of brain activation and functional connectivity. Hum. Brain Mapp..

[19] Buades-Rotger, M., Engelke, C. & Kraemer, U. M. Trait and state patterns of basolateral amygdala connectivity at rest are related to endogenous testosterone and aggression in healthy young women. *bioRxiv*, 248930 (2018). 10.1007/s11682-018-9884-2 29744800

[20] Schutter DJ, Honk JV (2004). Decoupling of midfrontal delta–beta oscillations after testosterone administration. Int. J. Psychophysiol..

[21] Schutter DJ, Peper JS, Koppeschaar HP, Kahn RS, van Honk J (2005). Administration of testosterone increases functional connectivity in a cortico-cortical depression circuit. The Journal of neuropsychiatry and clinical neurosciences.

[22] Westlye LT, Kaufmann T, Alnæs D, Hullstein IR, Bjørnebekk A (2017). Brain connectivity aberrations in anabolic-androgenic steroid users. Neuroimage: clinical.

[23] Heany, S. J. *et al*. Effects of Testosterone Administration on Threat and Escape Anticipation in the Orbitofrontal Cortex. *Psychoneuroendocrinology* (2018). 10.1016/j.psyneuen.2018.05.038 29902666

[24] Etkin A, Büchel C, Gross JJ (2015). The neural bases of emotion regulation. Nature Reviews Neuroscience.

[25] Gross JJ (2015). Emotion regulation: Current status and future prospects. Psychol. Inq..

[26] Panagiotidis D (2017). Exogenous testosterone in a non-social provocation paradigm potentiates anger but not behavioral aggression. Eur. Neuropsychopharmacol..

[27] Wagels Lisa, Radke Sina, Goerlich Katharina Sophia, Habel Ute, Votinov Mikhail (2017). Exogenous testosterone decreases men's personal distance in a social threat context. Hormones and Behavior.

[28] Patton JH, Stanford MS, Barratt ES (1995). Factor structure of the Barratt impulsiveness scale. J. Clin. Psychol..

[29] Buss AH, Perry M (1992). The aggression questionnaire. J. Pers. Soc. Psychol..

[30] Lilienfeld SO, Andrews BP (1996). Development and preliminary validation of a self-report measure of psychopathic personality traits in noncriminal population. J. Person. Assess..

[31] Gross JJ, John OP (2003). Individual differences in two emotion regulation processes: implications for affect, relationships, and well-being. J. Pers. Soc. Psychol..

[32] Watson D, Clark LA, Tellegen A (1988). Development and validation of brief measures of positive and negative affect: the PANAS scales. J. Pers. Soc. Psychol..

[33] Sjöberg RL (2008). A non-additive interaction of a functional MAO-A VNTR and testosterone predicts antisocial behavior. Neuropsychopharmacology.

[34] Chester DS (2015). Monoamine oxidase A (MAOA) genotype predicts greater aggression through impulsive reactivity to negative affect. Behav. Brain Res..

[35] Wagels, L. *et al*. Exogenous testosterone and the monoamine-oxidase A polymorphism influence anger, aggression and neural responses to provocation in males. *Neuropharmacology* (2019). 10.1016/j.neuropharm.2019.01.006 30639342

[36] Buckholtz JW (2008). Genetic variation in MAOA modulates ventromedial prefrontal circuitry mediating individual differences in human personality. Mol. Psychiatry.

[37] Yarkoni T, Poldrack RA, Nichols TE, Van Essen DC, Wager TD (2011). Large-scale automated synthesis of human functional neuroimaging data. Nat. Methods.

[38] Salimi-Khorshidi G (2014). Automatic denoising of functional MRI data: combining independent component analysis and hierarchical fusion of classifiers. NeuroImage.

[39] Griffanti L (2014). ICA-based artefact removal and accelerated fMRI acquisition for improved resting state network imaging. NeuroImage.

[40] Griffanti L (2016). Challenges in the reproducibility of clinical studies with resting state fMRI: An example in early Parkinson’s disease. NeuroImage.

[41] Ashburner J, Friston KJ (2005). Unified segmentation. NeuroImage.

[42] Marrelec G (2006). Partial correlation for functional brain interactivity investigation in functional MRI. NeuroImage.

[43] Friston KJ, Kahan J, Biswal B, Razi AA (2014). DCM for resting state fMRI. NeuroImage.

[44] Razi A, Kahan J, Rees G, Friston KJ (2015). Construct validation of a DCM for resting state fMRI. NeuroImage.

[45] Peper JS, van den Heuvel MP, Mandl RCW, Pol HEH, van Honk J (2011). in Psychoneuroendocrinology.

[46] Engman J, Linnman C, Van Dijk KRA, Milad MR (2016). Amygdala subnuclei resting-state functional connectivity sex and estrogen differences. Psychoneuroendocrinology.

[47] Kohn N (2014). Neural network of cognitive emotion regulation—an ALE meta-analysis and MACM analysis. NeuroImage.

[48] Frank D (2014). Emotion regulation: quantitative meta-analysis of functional activation and deactivation. Neurosci. Biobehav. Rev..

[49] Morawetz C, Bode S, Baudewig J, Kirilina E, Heekeren HR (2015). Changes in effective connectivity between dorsal and ventral prefrontal regions moderate emotion regulation. Cereb. Cortex.

[50] Langner, R., Leiberg, S., Hoffstaedter, F. & Eickhoff, S. B. Towards a human self-regulation system: Common and distinct neural signatures of emotional and behavioural control. *Neurosci. Biobehav. Rev*. (2018). 10.1016/j.neubiorev.2018.04.022 PMC5994341 29730485

[51] Höfer P, Lanzenberger R, Kasper S (2013). Testosterone in the brain: neuroimaging findings and the potential role for neuropsychopharmacology. Eur. Neuropsychopharmacol..

[52] Buhle JT (2014). Cognitive reappraisal of emotion: a meta-analysis of human neuroimaging studies. Cereb. Cortex.

[53] Banks SJ, Eddy KT, Angstadt M, Nathan PJ, Phan KL (2007). Amygdala–frontal connectivity during emotion regulation. Soc. Cogn. Affect. Neurosci..

[54] Kanske P, Heissler J, Schönfelder S, Bongers A, Wessa M (2010). How to regulate emotion? Neural networks for reappraisal and distraction. Cereb. Cortex.

[55] Votinov M, Aso T, Fukuyama H, Mima T (2016). A Neural Mechanism of Preference Shifting Under Zero Price Condition. Front. Hum. Neurosci..

[56] Votinov M, Pripfl J, Windischberger C, Sailer U, Lamm C (2015). Better you lose than I do: neural networks involved in winning and losing in a real time strictly competitive game. Sci. Rep..

[57] Bartra O, McGuire JT, Kable JW (2013). The valuation system: a coordinate-based meta-analysis of BOLD fMRI experiments examining neural correlates of subjective value. NeuroImage.

[58] Maier ME, Di Pellegrino G (2012). Impaired conflict adaptation in an emotional task context following rostral anterior cingulate cortex lesions in humans. J. Cognit. Neurosci..

[59] Hiser J, Koenigs M (2018). The multifaceted role of the ventromedial prefrontal cortex in emotion, decision making, social cognition, and psychopathology. Biol. Psychiatry.

[60] Pajer K (2006). Adrenal androgen and gonadal hormone levels in adolescent girls with conduct disorder. Psychoneuroendocrinology.

[61] Talih, F., Fattal, O. & Malone, J. D. Anabolic steroid abuse: psychiatric and physical costs. *Cleve. Clin. J. Med*. **74**, 341–344, 346, 349–352 (2007). 10.3949/ccjm.74.5.341 17506239

[62] McHenry J, Carrier N, Hull E, Kabbaj M (2014). Sex differences in anxiety and depression: role of testosterone. Front. Neuroendocrinol..

[63] Wainwright SR, Lieblich SE, Galea LA (2011). Hypogonadism predisposes males to the development of behavioural and neuroplastic depressive phenotypes. Psychoneuroendocrinology.

[64] Wu FC (2010). Identification of late-onset hypogonadism in middle-aged and elderly men. New Engl. J. Med..

[65] Seidman SN, Roose SP (2006). The sexual effects of testosterone replacement in depressed men: randomized, placebo-controlled clinical trial. J. Sex Marital Ther..

[66] Pope HG, Cohane GH, Kanayama G, Siegel AJ, Hudson JI (2003). Testosterone gel supplementation for men with refractory depression: a randomized, placebo-controlled trial. Am. J. Psychiatry.

[67] Bassil N, Alkaade S, Morley JE (2009). The benefits and risks of testosterone replacement therapy: a review. Ther. Clin. Risk Manag..

[68] Birn RM (2013). The effect of scan length on the reliability of resting-state fMRI connectivity estimates. NeuroImage.

[69] Dodhia S (2014). Modulation of resting-state amygdala-frontal functional connectivity by oxytocin in generalized social anxiety disorder. Neuropsychopharmacology.

