# Supplementary material for: Effects of exogenous testosterone application on network connectivity within emotion regulation systems
Source: Sci Rep. 2020 Feb 11;10:2352. doi: 10.1038/s41598-020-59329-0 (PMC7012825; doi:10.1038/s41598-020-59329-0)
Supplement: Supplementary file 1 — Supplementary data. [file 41598_2020_59329_MOESM1_ESM.docx]

**Effects of exogenous testosterone application on network connectivity within emotion regulation systems**

Mikhail Votinov, Lisa Wagels, Felix Hoffstaedter, Thilo Kellermann, Katharina S. Goerlich, Simon B. Eickhoff, Ute Habel

Link to the term emotion regulation at the Neurosynth. An automated meta-analysis of 161 studies

**http://www.neurosynth.org/analyses/terms/emotion%20regulation/**

*Genotyping*

Genotyping for the monoamine oxidase-A-linked polymorphism was performed primarily for analysis of the aggression and risk-taking related fMRI and further behavioral tasks, not reported here, but was included as an additional measure in this study. The genotype MAOA-L (long tandem repeats) and MAOA-S (short tandem repeats) were determined prior to the study and participants were randomly allocated to either the testosterone or the placebo group in order to keep a balanced group size. DNA from buccal mucosa cell samples was analyzed in a collaborate laboratory (Molecular Psychology, Ulm, Germany). While participants were randomly assigned to placebo or testosterone, they were stratified by the MAOA polymorphism. Experimenters were not aware of administration group or gene variant of the participants (for more details, see ([Wagels, et al., 2017a](#_ENREF_53)).

**Seed regions for ROI analysis.Supplementary Table 1.**

Coordinates of seed regions for ROI analysis.

| **Name** | **x** | **y** | **z** |
| --- | --- | --- | --- |
| *Left amygdala* | -18 | -4 | -16 |
| *Right amygdala* | 22 | -2 | -14 |
| *Left insula* | 38 | 20 | -4 |
| *Right insula* | -36 | 22 | -4 |
| *VMPFC* | 0 | 52 | -8 |
| *dACC/aMCC* | -2 | 22 | 28 |
| *PCC* | -2 | -54 | 28 |
| *Right DLPFC* | 34 | 26 | 40 |
| *Left DLPFC* | -36 | 16 | 40 |
| *SMA* | 2 | 20 | 46 |
| *Right IPL* | 52 | -48 | 44 |
| *Left IPL* | -46 | -50 | 44 |
| *Right VLPFC* | 48 | 18 | 26 |
| *Left VLPFC* | -48 | 16 | 26 |

Ventromedial prefrontal cortex (VMPFC), dorsal anterior cingulate cortex (dACC), ventral-lateral prefrontal cortex (VLPFC), dorsal-lateral prefrontal cortex (DLPFC), parietal cingulatecortex (PCC), supplementary motor area (SMA) and inferior parietal lobule (IPL).

**Hormonal results.Supplementary Table 2.**

In total, 96 participants underwent the resting state connectivity scanning. Serum levels in blood could not be assessed at point T3 for 6 participants and at T4 for 9 participants, due to .The hormonal level was significantly different between groups for T2, T3, and T4 points, but not at the baseline T1 measurement. The repeated measures ANCOVA revealed a main effect of Group F(1,83) = 21.69, p<0.001 and Time F(3,81) = 10.53, p<0.001 and an interaction of Time x Group F(3,81) = 14.38, p<0.001.

| **Time Points (T1)** | **Groups** | **Number of subjects** | **Testosterone Level**  **Mean ± SEM SD** | **P value** |
| --- | --- | --- | --- | --- |
| T1 | Placebo group | 45 | 16.64 ± 0.67 4.55 | p=0.81 |
|  | Testosterone group | 51 | 16.41 ± 0.64 4.63 |  |
| T2 | Placebo group | 45 | 15.06 ± 0.79 5.32 | p<0.001 |
|  | Testosterone group | 51 | 21.95 ± 1.12 7.99 |  |
| T3 | Placebo group | 42 | 15.17 ± 0.85 5.56 | p<0.001 |
|  | Testosterone group | 48 | 23.38 ± 1.24 8.61 |  |
| T4 | Placebo group | 42 | 14.25 ± 0.86 5.56 | p<0.001 |
|  | Testosterone group | 45 | 24.14 ± 1.42 9.55 |  |

**Questionnaires.Supplementary Table 3.**

| **Scales** | **Subscales** | **Placebo** | **Testosterone** | **P value** |
| --- | --- | --- | --- | --- |
| **Age** |  | 24.33 ± 3.63 | 24.15 ± 3.78 | .82 |
| **AQ** | *AQ Total* | 8.71 ± 1.61 | 9.03 ± 1.58 | .33 |
|  | *Anger* | 1.85 ± .50 | 1.96 ± .55 | .32 |
|  | *Hostility* | 2.02 ± .57 | 2.07 ± .49 | .68 |
|  | *Verbal* | 2.70 ± .48 | 2.76 ± .53 | .58 |
|  | *Physical* | 2.10 ± .61 | 2.23 ± .66 | .41 |
| **BIS11** | *Attentional* | 14.91 ± 2.93 | 15.70 ± 2.71 | .16 |
|  | *Motor* | 22.31 ± 3.29 | 21.77 ± 2.92 | .38 |
|  | *Non-planning* | 23.14 ± 3.62 | 24.61 ± 3.89 | .053 |
|  | *Total* | 60.37 ± 7.9 | 62.09 ± 5.42 | .2 |
| **PPI** | *CH* | 35.17 ± 4.39 | 35.92 ± 4.69 | .42 |
|  | *EG* | 42.26 ± 3.13 | 41.92 ± 3.41 | .61 |
|  | *AL* | 34.57 ± 4.07 | 34.84 ± 4.57 | .76 |
|  | *SI* | 44.77 ± 4.17 | 43.84 ± 6.27 | .39 |
|  | *SOI* | 45.51 ± 3.50 | 44.84 ± 4.17 | .40 |
|  | *FL* | 19.42 ± 2.69 | 20.15 ± 2.70 | .19 |
|  | *RA* | 77.53 ± 6.65 | 76.47 ± 6.32 | .42 |
|  | *O* | 31.37 ± 2.31 | 31.64 ± 2.23 | .56 |
|  | *I* | 109.71 ± 7.84 | 108.84 ± 10.26 | .64 |
|  | *II* | 195.77 ± 9.52 | 194.60 ± 12.15 | .60 |
|  | *Total* | 341.53 ± 13.65 | 339.53 ± 17.18 | .61 |
| **ERQ** | *Reappraisal* | 23.77±3.4 | 23.26±4.1 | .51 |
|  | *Suppression* | 16.95±2.4 | 17.64±3.5 | .28 |
| **PANAS** | *Positive Affect* | 2.83±0.1 | 2.77±0.06 | .65 |
|  | *Negative Affect* | 1.33±0.1 | 1.28±0.06 | .55 |
